# Surface-Oxidized Polymer-Stabilized Silver Nanoparticles as a Covering Component of Suture Materials

**DOI:** 10.3390/mi13071105

**Published:** 2022-07-14

**Authors:** Andrey Vladimirovich Blinov, Andrey Ashotovich Nagdalian, Sergey Nikolaevich Povetkin, Alexey Alekseevich Gvozdenko, Marina Nikolaevna Verevkina, Igor Vladimirovich Rzhepakovsky, Mariya Sergeevna Lopteva, David Guramievich Maglakelidze, Tatyana Semenovna Kataeva, Anastasiya Aleksandrovna Blinova, Alexey Borisovich Golik, Galina Vladimirovna Osipchuk, Mohammad Ali Shariati

**Affiliations:** 1 Department of Physics and Technology of Nanostructures and Materials, North Caucasus Federal University, 355017 Stavropol, Russia; ablinov@ncfu.ru (A.V.B.); d22003807-help@mail.ru (S.N.P.); gvozdenko.1999a@gmail.com (A.A.G.); 78igorr@mail.ru (I.V.R.); ogoniock2015@mail.ru (D.G.M.); nastya_bogdanova_88@mail.ru (A.A.B.); lexgooldman@gmail.com (A.B.G.); 2 Saint Petersburg State Agrarian University, 196601 Saint Petersburg, Russia; 3 Department of Parasitology and Veterinary Examination, Anatomy and Pathanatomy Named after Professor S. N. Nikolsky, Stavropol State Agrarian University, 355017 Stavropol, Russia; serenkiy7@yandex.ru; 4 Veterinary Medicine Laboratory of the Animal Husbandry, Veterinary Medicine Department, North Caucasus Federal Agrarian Research Centre, 356241 Mikhailovsk, Russia; zmejka-2007@mail.ru; 5 Department of Parasitology, Veterinary and Sanitary Expertise, Faculty of Veterinary Medicine, Kuban State Agrarian University Named after I. T. Trubilin, 350044 Krasnodar, Russia; cluster26@yandex.ru; 6 Laboratory of Embryos Reproduction and Transplantation, Scientific-Practical Institute of Biotechnologies in Animal Science and Veterinary Medicine, 249053 Maksimovka Village, Moldova; galadok@rambler.ru; 7 Department of Scientific Research, K.G. Razumovsky Moscow State University of Technologies and Management, 109004 Moscow, Russia; shariatymohammadali@gmail.com

**Keywords:** silver nanoparticles, silver oxide nanoparticles, polyvinylpyrrolidone, suture material, oxidation, surface plasmon resonance

## Abstract

In this work, we obtained silver nanoparticles stabilized with polyvinylpyrrolidone, ranging in size from 70 to 110 nm, which exhibits good crystallinity and anisotropic structure. For the first time, we studied the influence of the molar ratio of silver between silver and peroxide on the oxidation process of the nanoparticles and determined the regularities of this process by analyzing changes in absorption spectra. Our results showed that at molar ratios of Ag:H_2_O_2_ = 1:1 and 1:5, dependences of changes in the intensity, position and half-width of the absorption band of the plasmon resonance are rectilinear. In vivo studies of silver nanoparticles have shown that silver nanoparticles belong to the toxicity class III (moderately hazardous substance) and to the third group according to the degree of accumulation. We established that silver nanoparticles and oxidized silver nanoparticles form a uniform layer on the surface of the suture material. We found that the use of the suture material with silver nanoparticles and oxidized silver nanoparticles does not cause allergic reactions in the organisms of laboratory animals.

## 1. Introduction

Despite significant medical development, in some cases, secondary patient infection occurs during surgical interventions. Bellows et al. (2011) noted that the incidence of infections after colorectal surgery remains at 26%, resulting in longer hospital stays, delayed initiation of adjuvant therapy and higher health care costs [1]. The authors of [2] wrote that the frequency of infections after spinal surgery is about 12%, depending on the diagnosis and complexity of the procedure. The causes of infection of postoperative wounds and sutures may be insufficient sanitation of the surgical tools or room or weakened immunity of the patient, which leads to the development of pathogens in the human body, etc.

Martino et al. (2019) declared that postoperative infections lead to the aggravation of the underlying disease course and to secondary suture ruptures, which poses a threat to the life of the patient [3]. In this regard, the development of preventive methods to combat postoperative infections is a promising task of modern healthcare. One of the preventive methods to combat postoperative infections is to condition the suture material with substances with highly antibacterial and antifungal activity.

Silver nanoparticles (Ag NPs) are a strong antibacterial and antifungal agent that inhibits the growth of Gram-positive (*Bacillus cereus*, *Staphylococcus aureus*, *Micrococcus luteus*, *Bacillus subtilis*, *Enterococcus species*) and Gram-negative (*Pseudomonas aeruginosa*, *Salmonella typhi*, *Escherichia coli*, *Klebsiella pneumonia*) bacteria, as well as pathogenic and conditionally pathogenic microorganisms (*Candida albicans*, *Sclerotinia homoeocarpa*, *Macrophomina phaseolina*) [4,5,6,7,8,9].

Ag NPs have a number of important advantages over other antibacterial drugs, including a lack of microorganism tolerance or virus resistance and safety to the human body [10,11,12]. This fact opens up prospects for the use of Ag NPs in various fields of science and technology: for the prevention and control of infectious processes, including antiseptic washing, water disinfection, creation of antibacterial films for food, antimicrobial protection of clothing, shoes, household items and also during general cleaning in medical-preventive and penitentiary institutions, municipal service facilities, public catering enterprises, in sports complexes and other high-attendance organizations and much more [13,14,15,16,17,18].

It is known that the mechanism of antibacterial activity of Ag NPs is based on the release of silver ions (Ag^+^). Ag NPs located in the vicinity of a bacterial cell or adsorbed on its surface are capable of releasing tens of thousands of Ag atoms, creating a locally high concentration of antibacterial ions, which leads to the dissolution of the bacterial cell wall [19,20,21,22,23,24,25,26,27,28].

In the works [24,29,30] there is information about the high bactericidal properties of silver oxide (Ag_2_O) or surface-oxidized Ag NPs. In [28], the authors synthesized Ag_2_O NPs using *Ficus benghalensis* root extract. It was found that the obtained Ag_2_O NPs exhibited high antibacterial activity, which exceeds the antibacterial activity of Ag NPs against caries bacteria by 20%. In [31] it is noted that Ag_2_O NPs are safe for the human body. In this regard, the aim of this work is to develop a technique for the synthesis of Ag NPs and oxidized Ag NPs and to study the possibility of their use as a coating component of suture materials.

## 2. Materials and Methods

### 2.1. Substances and Reagents

Silver nitrate (“TD UZHR” LLC, Moscow, Russia), polyvinylpyrrolidone (PVP) (“AK Sintvita” LLC, Kursk, Russia), sodium hydroxide (“Povolzhye” LLC, Dzerzhinsk, Russia), ethyl alcohol (“Merck KGaA”, Darmstadt, Germany), phosphoric acid (“LenReaktiv” Inc., Saints Petersburg, Russia), acetic acid (“LenReaktiv” Inc., Saints Petersburg, Russia), boric acid (“LenReaktiv” Inc., Saints Petersburg, Russia), sodium chloride (“LenReaktiv” Inc., Saints Petersburg, Russia) and hydrogen peroxide (“Khimprom” Inc., Novocheboksarsk, Russia) were used.

### 2.2. Method of Synthesis of Ag NPs Stabilized with PVP

First, 12.5 g of PVP was weighed on analytical scales. They were then quantitatively transferred to a 100 mL chemical reactor and dissolved in 38 mL aqueous alcohol solution. The mixture was stirred for 30 min at t = 60 °C. A silver nitrate (AgNO_3_) solution was prepared separately; for this purpose, 0.385 g of AgNO_3_ was weighed on analytical scales and quantitatively transferred to a beaker. Then it was dissolved in 1.2 mL of distilled water. AgNO_3_ solution was added to the PVP dissolved in an aqueous alcohol solution dropwise and mixed intensively at t = 60 °C for 60 min. The resulting sol had a yellow–green color.

### 2.3. Methods

Sample micrographs and elemental composition data were obtained using a scanning electron microscope MIRA3-LMH (Tescan, Brno-Kohoutovice, Czech Republic) with a system for determining the elemental composition AZtecEnergy Standard/X-max 20 (standard). The sample preparation was carried out as follows: a double-sided conductive carbon tape was glued on a standard instrumentation table (12 mm). Then, Ag NPs powder was applied on the conductive carbon tape. Finally, a carbon coating with a thickness of about 10 nm was deposited.

The parameters of the measurement were as follows:

- Voltage: 10 kV.
- Work distance: 4.9 mm.
- In-Beam secondary electron detector.

The phase composition of the Ag NPs was investigated by X-ray diffraction analysis on an Empyrean diffractometer (PANalytical, Almeo, The Netherlands). The following measuring parameters were used:

- Copper cathode (wavelength 1.54 Å).
- Measurement range: 10–90 2θ°.
- Sampling frequency: 0.01 2θ°.

To study functional groups in the obtained samples, IR spectroscopy was used. The IR spectra were recorded on an FSM-1201 IR spectrometer with Fourier transform. The measurement range was 400–4400 cm^−1^.

The determination of the average hydrodynamic radius of the particles was carried out by photon correlation spectroscopy on the Photocor-Complex device (“Antek-97” LLC, Moscow, Russia). The computer processing of the results was carried out using DynaLS V.2 software (“Fotokor“ LLC, Moscow, Russia) [32].

The molecular simulation was carried out in the IQmol 2.13.0 molecular editor (Q-Chem Inc., Pleasanton, CA, USA), and the quantum-chemical calculations of the models were carried out using the QChem 5.0 software (Q-Chem Inc., Pleasanton, CA, USA) with the following parameters: Calculation—Energy, Method—HF, Basis—6-31G, Convergence—5 and Force field—Chemical. IR spectra were calculated in the range of 400 to 4000 cm^−1^ with the following parameters: Calculation—Frequency, Method—HF, Basis-–6-31G, Convergence—5, Force field-–Chemical [33,34,35].

### 2.4. Investigation of the Aggregative Stability of Ag NPs

To study the aggregative stability of Ag NPs, 2 series of solutions were prepared: the first series—with different ionic strength, the second series—with different active acidity of the medium. Sodium chloride was used to prepare solutions with different ionic strengths. The ionic strength of the solution varied from 0.5 to 3.5. To prepare solutions with a different active acidity (pH) of the medium, we used 0.04 M solution of boric, citric and orthophosphoric acids and 0.2 M solution of sodium hydroxide. Therefore, we prepared solutions with pH = 2; 5; 7; 9 and 12 [36].

Dried samples of Ag NPs were dissolved in prepared solutions and kept under these conditions for 1 h. Then, absorption spectra were performed. The initial solutions without Ag NPs were used as comparison solutions. Absorption spectra were taken on an SF-56 optical spectrophotometer (OKB “Spectrum”, Saints Petersburg, Russia).

### 2.5. Investigation of the Oxidation Process of Ag NPs

To study the oxidation process, various amounts of hydrogen peroxide (H_2_O_2_) were added to the preparation of Ag NPs. We prepared 5 series: Ag:H_2_O_2_ = 1:1; Ag:H_2_O_2_ = 1:5; Ag:H_2_O_2_ = 1:10; Ag:H_2_O_2_ = 1:15 and Ag:H_2_O_2_ = 1:20. After mixing the Ag NPs and H_2_O_2_ preparations, we measured the absorption spectra. The measurements were carried out for 80 min. The absorption spectra were taken on an SF-56 optical spectrophotometer (OKB “Spectrum”, Saints Petersburg, Russia).

### 2.6. In Vivo Studies of Oxidized Ag NPs

To study the acute toxicity and cumulative effect of oxidized Ag NPs, we used laboratory white mice BALB/c (age—from 4 to 5 weeks). The laboratory animals were kept and placed in a pathogen-protected room with controlled temperature and humidity. The research was conducted in the vivarium of Stavropol State Medical University (Stavropol, Russia) in accordance with the recommendations set forth in the “Guidelines for experimental (preclinical) study of new pharmacological substances” [37].

Sixty laboratory white mice were divided into 6 groups with ten individuals each. The first group served as a control, and the laboratory mice of the second, third, fourth, fifth and sixth groups received the developed orally in increasing doses from 4000 to 4500 mg/g. To study the cumulative effect, we formed 2 groups of laboratory white mice. In each group were 10 individuals with masses ranging from 20 to 22 g. The preparation of oxidized Ag NPs stabilized with PVP was administered orally to the laboratory animals of the first group once a day.

The second group of animals served as a control. Distilled water was used as a comparison liquid, corresponding in volume to the amount of Ag NPs preparation injected into the mice of the first group.

### 2.7. Treatment of Suture Material with Ag NPs and Oxidized Ag NPs

To form a layer of Ag NPs and oxidized Ag NPs on the surface of the suture material, the synthesis of Ag NPs and oxidized Ag NPs was carried out in the presence of the suture material. Then, the suture material was kept in the resulting solution for 24 h and extracted and dried at room temperature for 48 h.

### 2.8. In Vivo Test of Suture Material

To test the suture material, we conducted studies on 6-month-old Californian breed rabbits (*Sylvilagus bachmani*). The laboratory animals were kept and placed in a pathogen-predicted room with controlled temperature and humidity. The research was carried out on the basis of the Faculty of Medicine and Biology of North Caucasus Federal University in accordance with the recommendations set forth in the “Guidelines for experimental (preclinical) study of new pharmacological substances” [37].

Three groups of laboratory animals of 3 individuals were formed. For the animals of the first group, we used suture material treated with oxidized Ag NPs, for the second group—suture material treated with Ag NPs, and for the third group—untreated suture material. For the experiment, an incision was made along the white line of the animal’s abdomen. We applied a cruciform suture capturing the muscle, fat and serous layers of the abdominal wall. Nylon thread was used as a suture material. After the operation, the laboratory animals were placed back in the chamber.

### 2.9. Histological Research

For histological research, sections of the abdominal wall in the area of the sutures were taken on the 10th day of wound healing. Under anesthesia, a section of the postoperative wound was cut with the underlying muscles and placed in a 10% formalin solution. Histological sections with a thickness of 5–6 μm were made on a sledge microtome MS-2 (“ATMpractica” LLC, Saints Petersburg, Russia). Finished sections were stained with hematoxylin and eosin, followed by histopathological analysis. Histological micro-preparation was evaluated using a laboratory microscope of the Axio Imager 2 (A2) research class (Carl Zeiss Microscopy, Oberkochen, Germany) at magnifications of ×50, ×100 and ×200 with image fixation using a specialized AxioCam MRc5 camera (Carl Zeiss Microscopy) and Zen V.2 software (Carl Zeiss Microscopy) [38].

## 3. Results and Discussion

### 3.1. Investigation of Ag NPs

**Figure 1:**
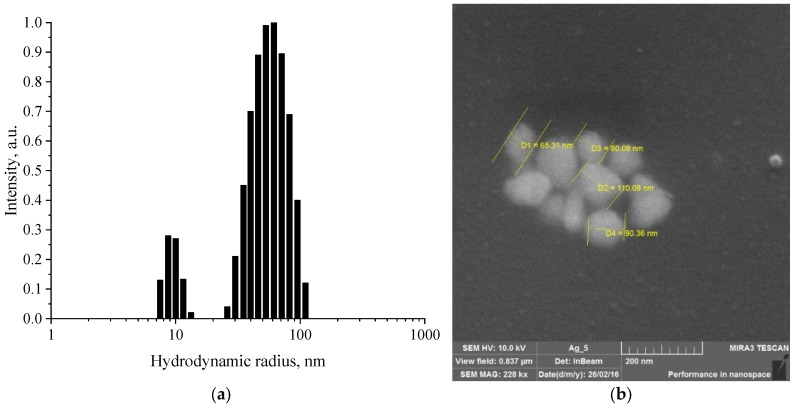
Histogram of Ag NPs hydrodynamic radius distribution (**a**) and SEM micrograph of a dried sample of Ag NPs (**b**).

At the first stage, we examined a sample of Ag NPs using the dynamic light scattering (DLS) method and SEM. The resulting histogram of the hydrodynamic radius distribution and SEM-micrography of the sample of Ag NPs are shown in Figure 1.

As can be seen from Figure 1a, the sample had a bimodal distribution: the average hydrodynamic radius of the first fraction was 10 nm, and that of the second was 60 nm. This fact assumes the presence in the sample of either two fractions of particles of appropriate sizes or particles of anisotropic shape. Figure 1b shows that the dried samples consist of individual nanoparticles with sizes from 50 to 110 nm, having a non-spherical shape.

**Figure 2:**
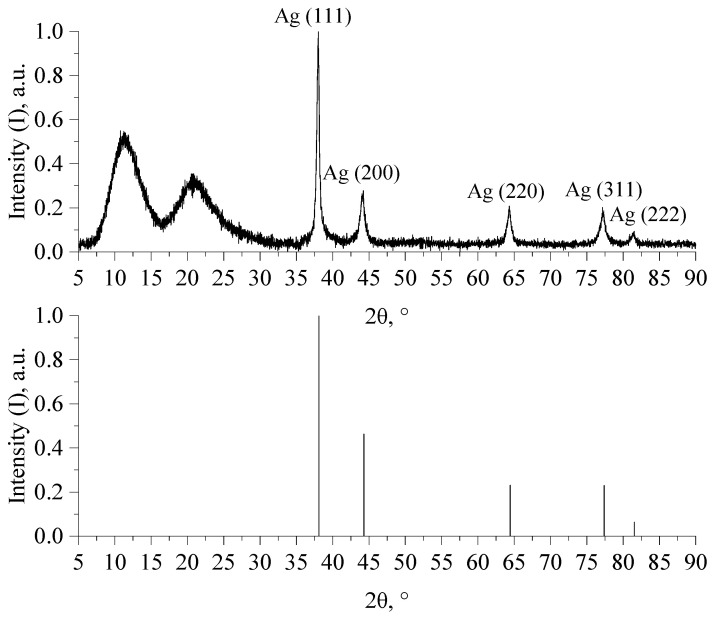
Diffractogram of a dried sample of Ag NPs.

In order to study the morphology and structure of the particles, samples of Ag NPs were dried by spray drying. The dried samples were examined by X-ray diffraction analysis (XRD) and scanning electron microscopy (SEM). The resulting diffractogram is shown in Figure 2.

Diffractogram analysis showed that silver occurs as nanocrystallites with a cubic face-centered crystal lattice, the spatial group Fm-3m, with unit cell parameters a = b = c = 0.409 nm. Wide bands in the area from 2θ = 8° to 2θ = 17° and from 2θ = 17° to 2θ = 25° correspond to PVP, which was used as a stabilizer of nanoparticles.

**Figure 3:**
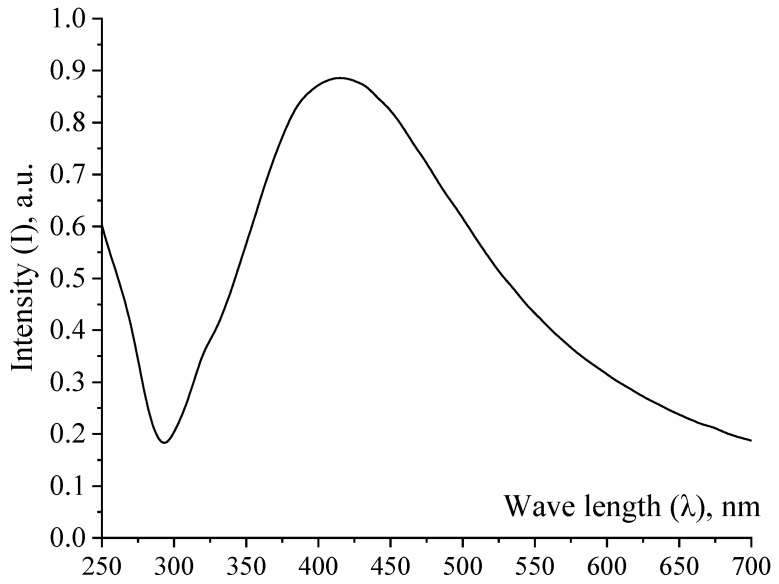
Absorption spectrum of Ag NPs sample.

At the next stage of the research, a sample of Ag NPs was examined by spectrophotometry on the SF-56 spectrophotometer (OKB “Spectrum” LLC, Saints Petersburg, Russia). The absorption spectrum is shown in Figure 3.

Analysis of the absorption spectrum of Ag NPs in the UV and visible regions showed the presence of a band in the spectrum with an absorption maximum at 425 nm, which corresponds to the surface plasmon resonance of Ag NPs [39,40].

**Figure 4:**
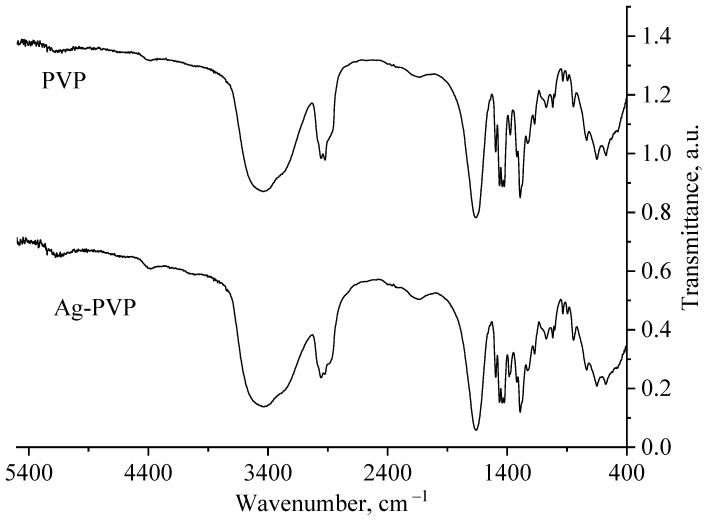
IR transmission spectra of PVP and a preparation of Ag NPs stabilized by PVP.

**Figure 5:**
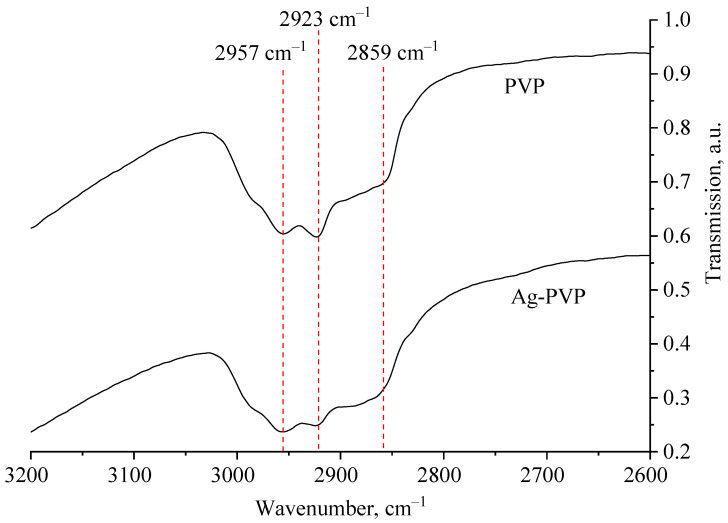
Section of IR transmission spectra of PVP and a preparation of Ag NPs stabilized by PVP.

At the next stage of research, the stabilization process of Ag NPs with PVP was considered. For this purpose, computer quantum chemical modeling of the interaction of Ag NPs with the PVP and IR spectroscopy of Ag NPs samples were carried out. IR spectra of a sample of Ag NPs stabilized with PVP and a sample of pure PVP are shown in Figure 4 and Figure 5.

Analysis of Figure 5 showed that all deformation fluctuations in the transmission spectra of PVP and Ag NPs-PVP coincide. Deviations were found in the region of valence oscillations of N-H bonds at frequencies from 2800 to 3000 cm^−1^ (Figure 5), which is associated with the adsorption of PVP during Ag NPs stabilization.

**Figure 6:**
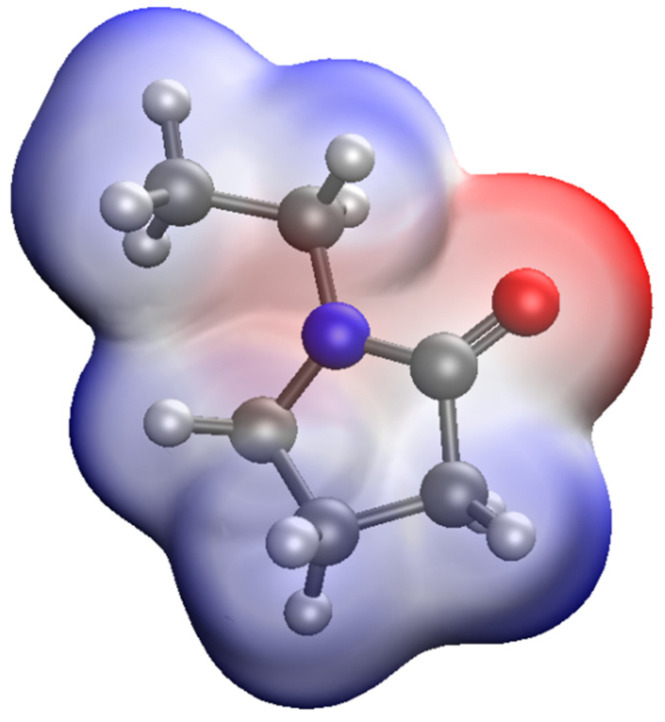
Model of PVP molecule with an electron density distribution.

**Figure 7:**
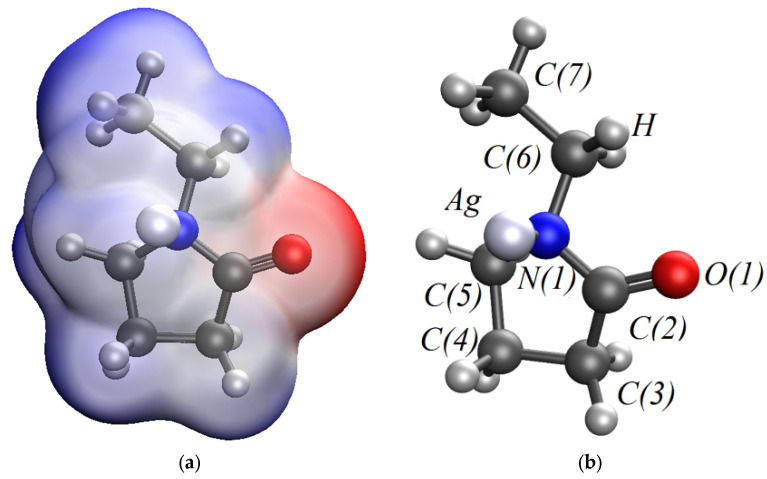

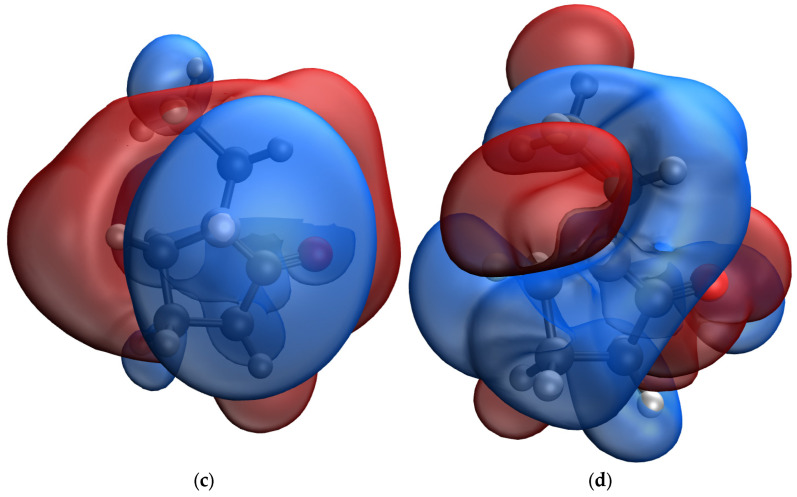
A model of the interaction of Ag atom with a PVP monomer unit through nitrogen: (**a**)—the electron density distribution, (**b**)—the Ag-PVP molecular complex model, (**c**)—the model of the molecular orbital HOMO (highest occupied molecular orbital) and (**d**)—the model of the molecular orbital LUMO (lowest unoccupied molecular orbital).

**Figure 8:**
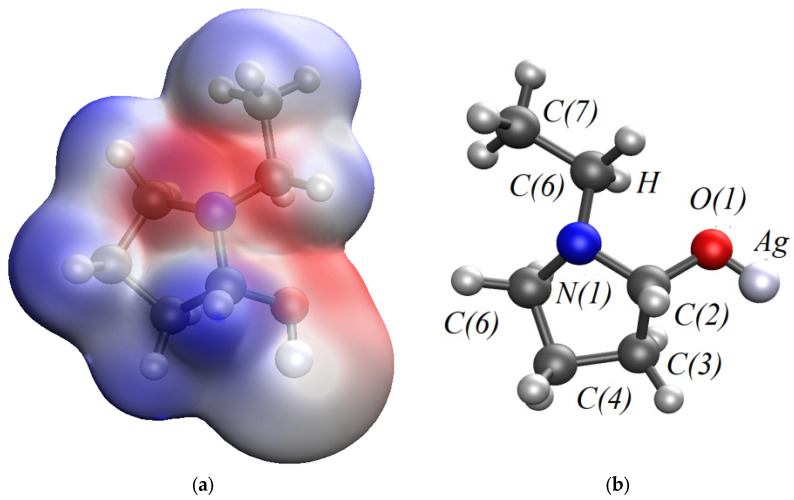

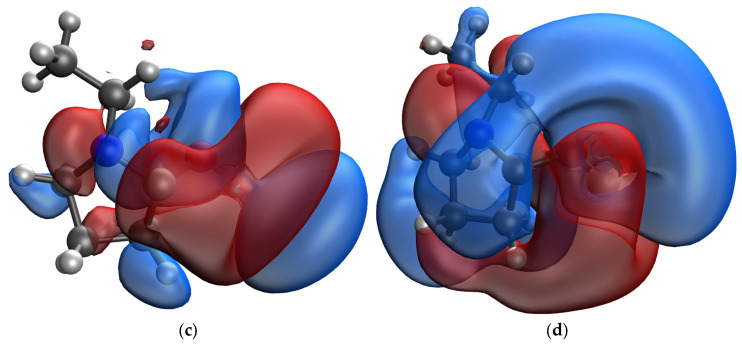
A model of the interaction of Ag atom with a PVP monomer unit through oxygen: (**a**)—the electron density distribution, (**b**)—the model of Ag-PVP molecular complex, (**c**)—the model of the molecular orbital HOMO (highest occupied molecular orbital) and (**d**)—the model of the molecular orbital LUMO (lowest unoccupied molecular orbital).

In the quantum chemical modeling of the stabilization of Ag NPs with PVP, the interaction of the Ag atom and the monomer bond of PVP via nitrogen and oxygen was considered (Figure 6, Figure 7 and Figure 8).

Based on quantum chemical calculations, we determined the interaction energy, the values of the HOMO and LUMO molecular orbitals, and the value of the chemical rigidity of the system. The obtained data are presented in Table 1.

As a result, the Ag-PVP system has a lower energy (E = −5533.5474 kcal/mol) when a bond is formed between a nitrogen atom in the PVP molecule and Ag atom than when a bond is formed between oxygen in the PVP molecule and Ag atom (E = −5532.8331 kcal/mol). It is shown that the chemical rigidity of the system in the case of the interaction of a Ag atom with a PVP monomer unit via nitrogen (*η* = 0.085) is higher than in the case of interaction of a Ag atom with the PVP monomer unit via oxygen (*η* = 0.073), which indicates that the molecular system formed by nitrogen is more stable [41,42].

**Figure 9:**
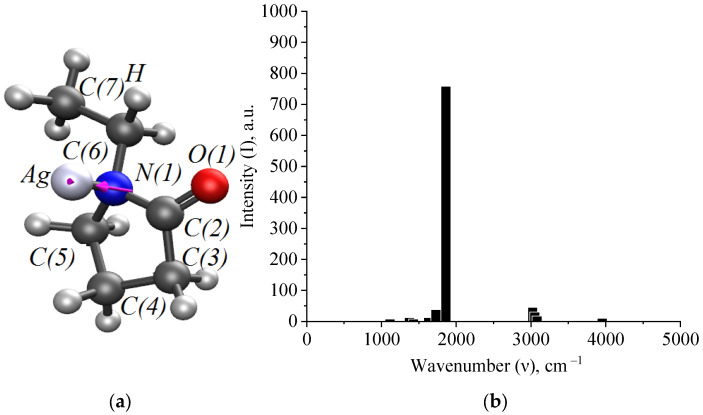
Modeling of spectral characteristics of Ag-PVP molecular complex (interaction through nitrogen): (**a**)—the oscillation of the N-Ag bond and (**b**)—the IR spectrum.

**Figure 10:**
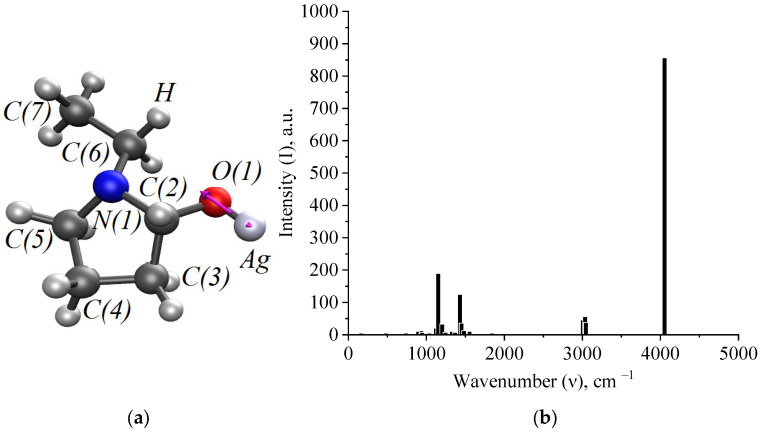
Modeling of spectral characteristics of Ag-PVP molecular complex (interaction through oxygen): (**a**)—the oscillation of the O-Ag bond and (**b**)—the IR spectrum.

At the next stage of modeling, we obtained spectral characteristics of Ag-PVP molecular complexes formed through nitrogen and oxygen. The results are presented in Figure 9 and Figure 10.

Thus, analysis of the generated IR spectra of Ag-PVP molecular complexes showed a high-intensity band of the valence oscillation of the O-Ag bond and a low-intensity band of the valence oscillation of the bond. Comparison of the data obtained by simulation with the IR spectrum obtained in the practice of Ag NPs stabilized with PVP (Figure 4 and Figure 5) showed the absence of a high-intensity band at 4000 cm^−1^, indicating the interaction of PVP molecules with the surface of Ag NPs by forming a N-Ag chemical bond.

### 3.2. Investigation of Aggregative Stability and the Oxidation Process of Ag NPs

**Figure 11:**
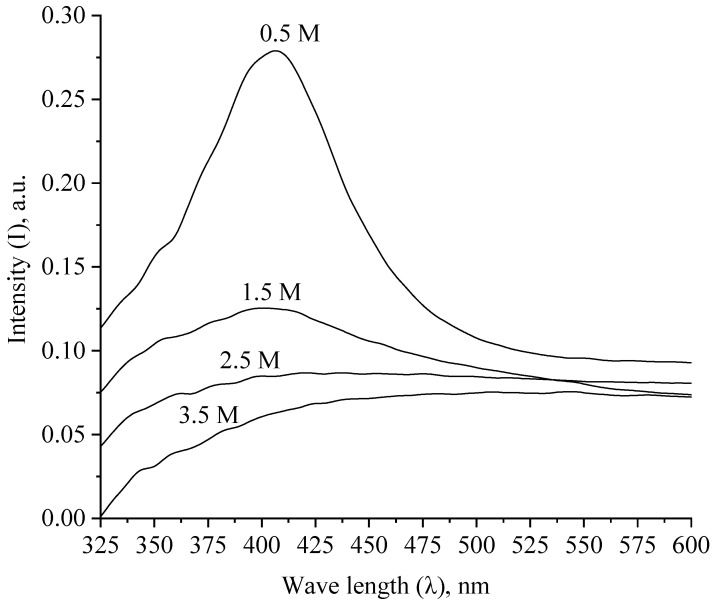
Absorption spectra of Ag NPs with different values of ionic strengths of solutions.

**Figure 12:**
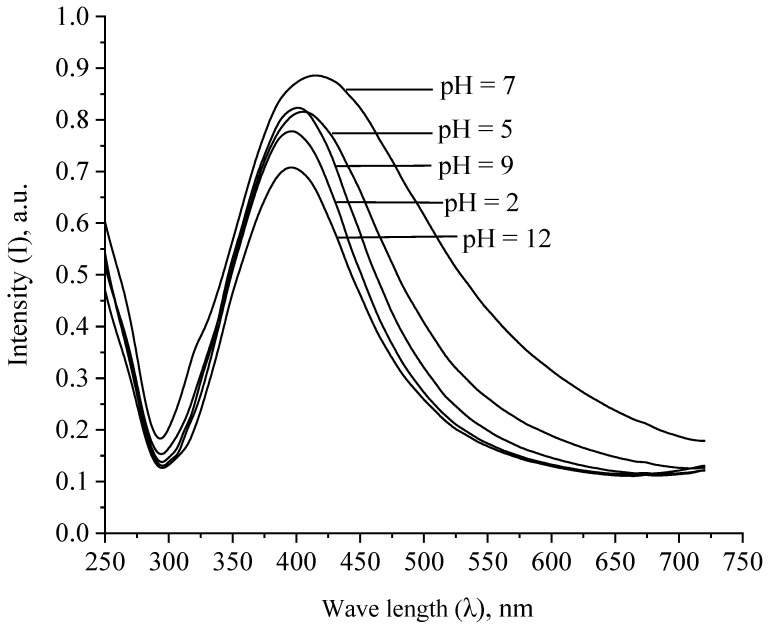
Absorption spectra of Ag NPs with different values of pH of the medium.

At the next stage, we investigated the aggregative stability of Ag NPs when the ionic strength and pH of the solution changed. The obtained spectra are shown in Figure 11 and Figure 12.

As shown by the analysis of the spectra presented in Figure 11, the intensity of the surface plasmon resonance band decreases with an increase in the ionic strength of the solution, which is associated with an increase in the size of Ag NPs due to electrostatic coagulation under the action of Cl^−^ ions. Absorption at λ = 410 nm was not observed in samples with an ionic strength of more than 1.5, which indicates the aggregation of all Ag NPs.

As shown by the analysis of Figure 12, the greatest changes in the absorption spectra of Ag NPs are observed in the strongly acidic (pH = 2) and strongly alkaline (pH = 12) pH region. However, it is important to note that we observed an absorption band characteristic of Ag NPs in all samples. This fact indicates a high aggregative stability of Ag NPs stabilized by PVP in the entire pH range.

**Figure 13:**
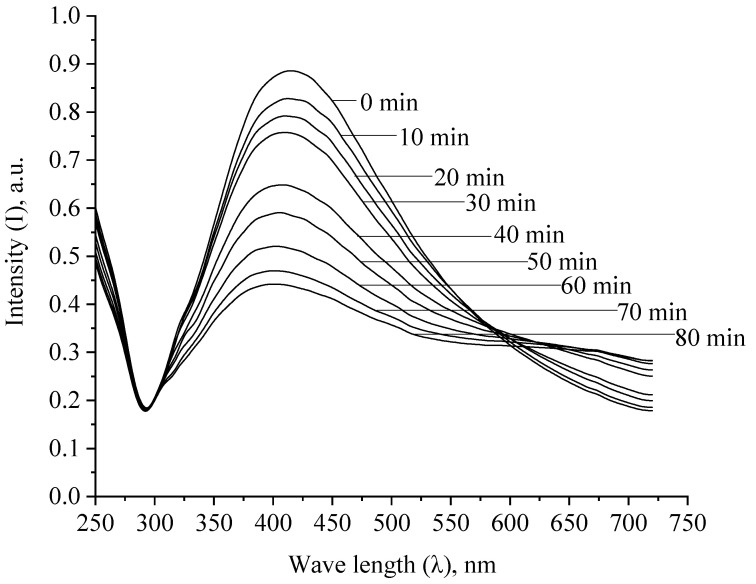
Absorption spectra of a mixture of Ag NPs and an oxidizer in the ratio Ag:H_2_O_2_ = 1:1.

Next, we studied the oxidation process of Ag NPs. The obtained spectral dependences are presented in Figure 13 and in the Supplementary Materials.

**Figure 14:**
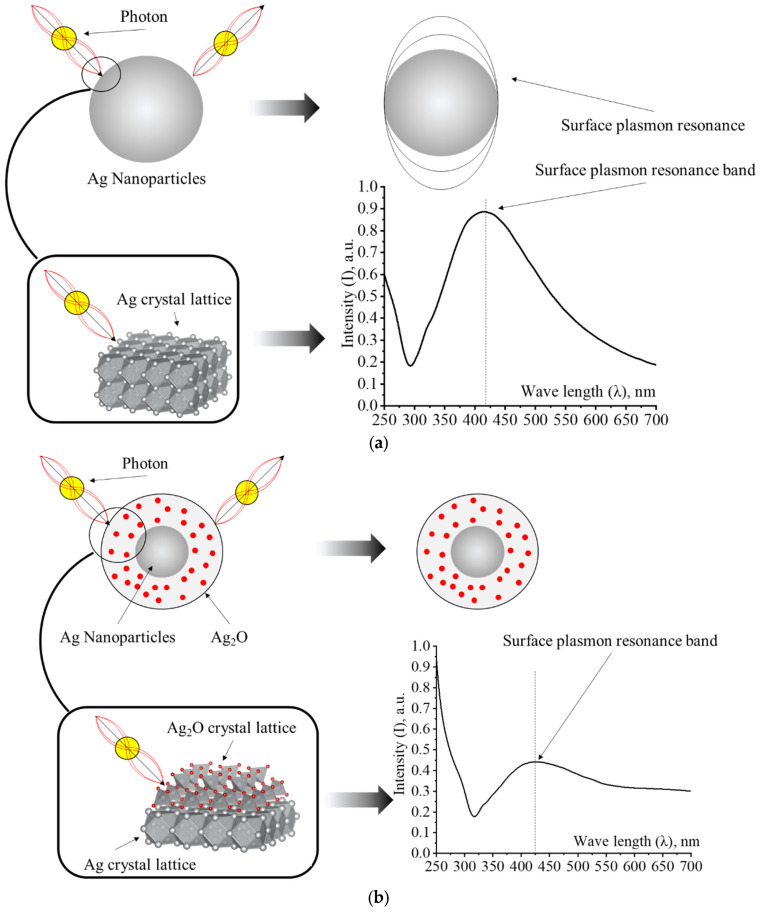
Diagram of changes in optical properties during oxidation of Ag NPs: (**a**)—Ag NPs and (**b**)—oxidation of Ag NPs surface.

By adding H_2_O_2_ to the Ag NPs samples, we observed a gradual discoloration of sols and a decrease in the intensity of the plasmon resonance band. These changes are associated with the oxidation of the surface of Ag NPs and the loss of their optical properties—the surface plasmon resonance. As it is known, plasmon resonance is an absorption caused by the coincidence of the frequencies of incident radiation and the frequency of the electron gas oscillation in the surface layer of particles [43,44]. Therefore, this absorption can only be detected in metallic particles. Thus, during oxidation, the surface layer of the Ag NPs preparation loses its metallic nature due to the formation of silver oxides and hydroxides on the surface of nanoparticles (Figure 14).

**Figure 15:**
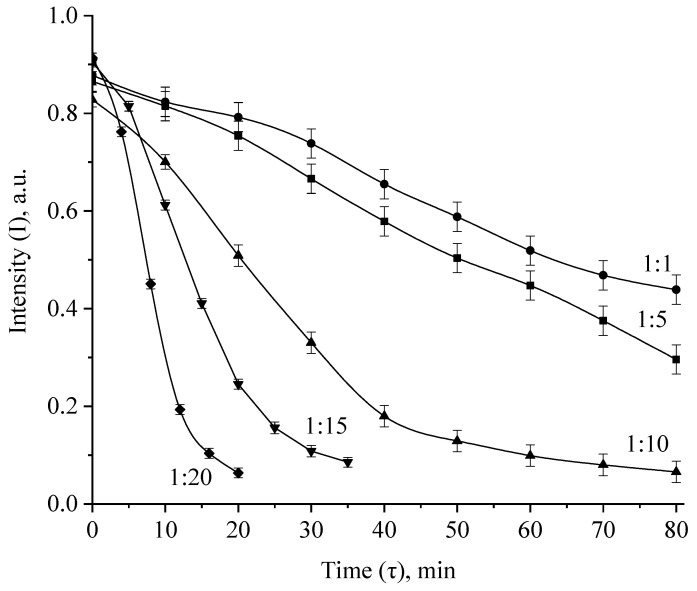
Kinetic dependences of changes in the intensity of the absorption band of the plasmon resonance of Ag NPs during oxidation with H_2_O_2_.

**Figure 16:**
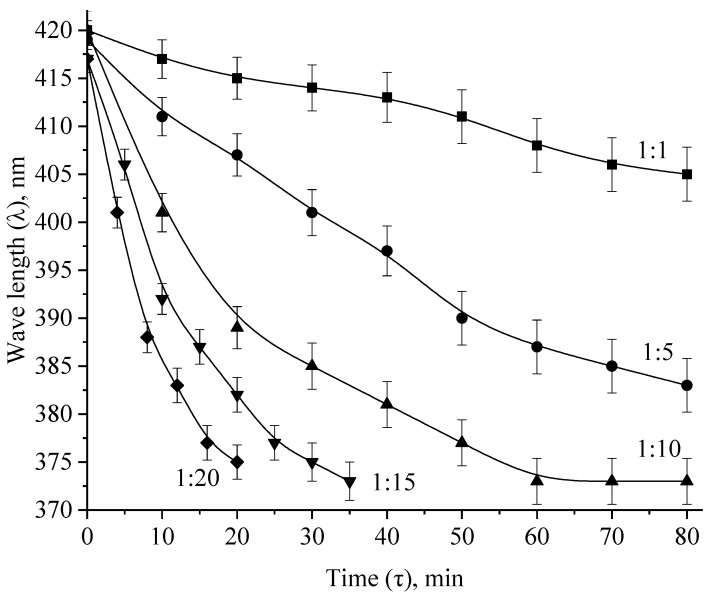
Kinetic dependences of changes in the position of the absorption band of the plasmon resonance of Ag NPs during oxidation with H_2_O_2_.

Analysis of the spectra presented in Figure 13 and in the Supplementary Materials allowed us to construct kinetic dependences of changes in the intensity of the plasmon resonance band, the position of the plasmon resonance band and the half-width of the plasmon resonance band on time, which are presented in Figure 15 and Figure 16 and in the Supplementary Materials.

The analysis of the obtained data showed that at Ag:H_2_O_2_ = 1:1 and 1:5 ratio, the dependencies of the changes in the intensity, position and half-width of the absorption band of the plasmon resonance are rectilinear, and complete discoloration after 80 min of exposure was not observed. At molar ratios Ag:H_2_O_2_ = 1:10, 1:15 and 1:20, the intensity dependences the plasmon resonance absorption band of Ag NPs took the form of exponents, and the complete discoloration of sols was observed after 20 min, 30 min and 40 min, respectively.

### 3.3. In Vivo Studies of Oxidized Ag NPs

At the next stage of research, we evaluated the acute toxicity of oxidized Ag NPs. The following toxicity parameters were determined: the minimum suppressive dose (MSD), the average lethal dose (LD_50_), the doses of the effect of LD_16_ and LD_84_ of Ag NPs preparation and the error index of the average dose of the effect (SLD_50_), which are presented in Table 2.

The test results allow to classify the oxidized Ag NPs stabilized with PVP as a toxicity class III preparation (moderately dangerous substance) according to the Russian State Standard GOST 12.1.007-76 SSBT, “Harmful substances. Classification and general safety requirements” [45].

Then, we conducted an experiment on accelerated determination of the cumulative effect. Based on the results of the conducted experiment on the accelerated determination of the cumulation coefficient, the preparation of the oxidized Ag NPs stabilized with PVP can be attributed by the degree of cumulation to group 3 (substances with moderate cumulation) [45].

### 3.4. Investigation of Ag NPs and the Oxidized Ag NPs as a Covering Component of Suture Materials

**Figure 17:**
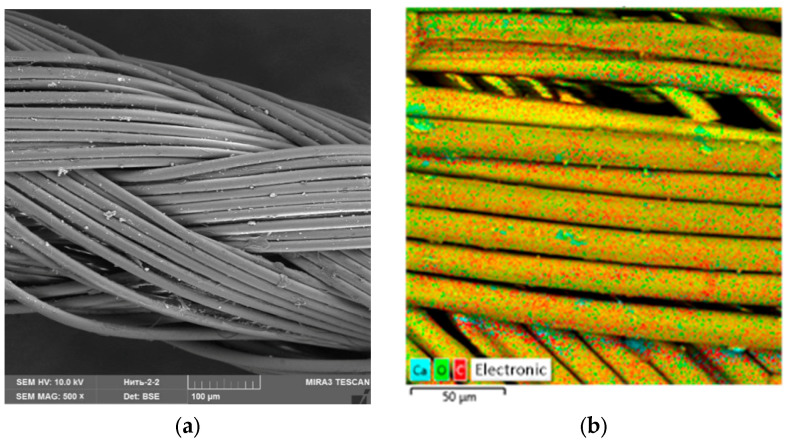

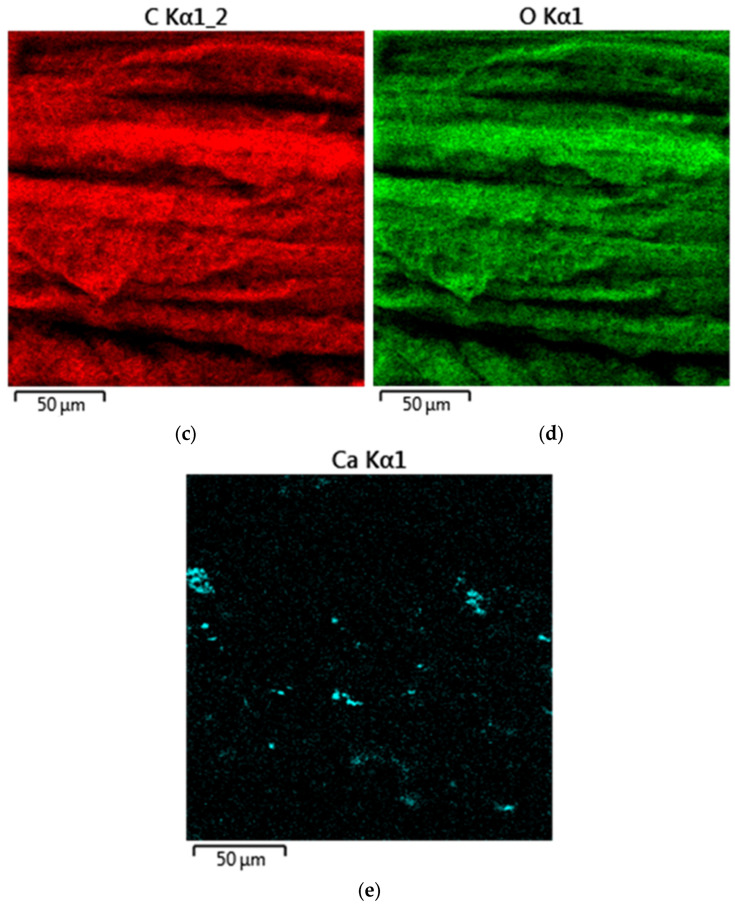
Control sample of suture material: (**a**)—SEM micrograph, (**b**)—multilayer map of EDS, (**c**)—elemental map of EDS (C), (**d**)—elemental map of EDS (O) and (**e**)—elemental map of EDS (Ca).

**Figure 18:**
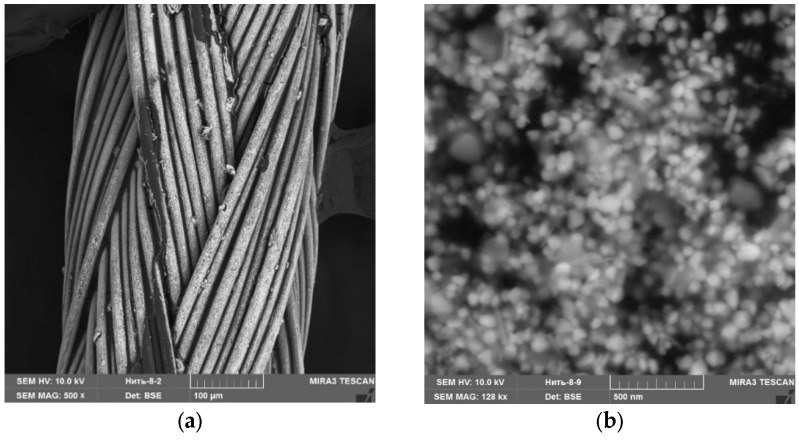

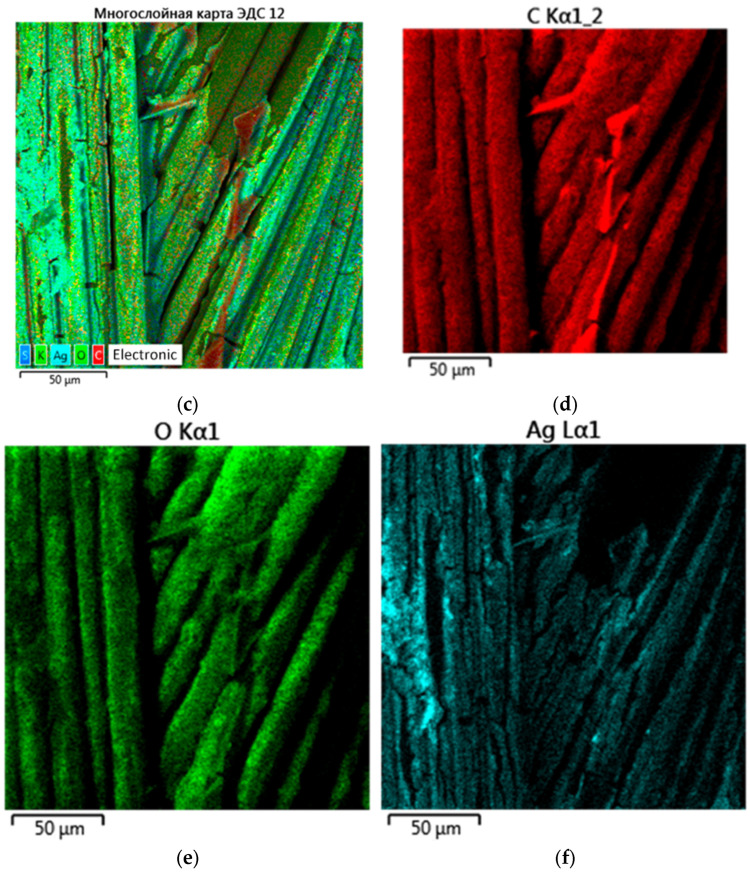
A sample of suture material treated by oxidized Ag NPs: (**a**)—SEM micrograph (×500), (**b**)—SEM micrograph (×128,000), (**c**)—multilayer map of EDS, (**d**)—elemental map of EDS (C), (**e**)—elemental map of EDS (O) and (**f**)—elemental map of EDS (Ag).

**Figure 19:**
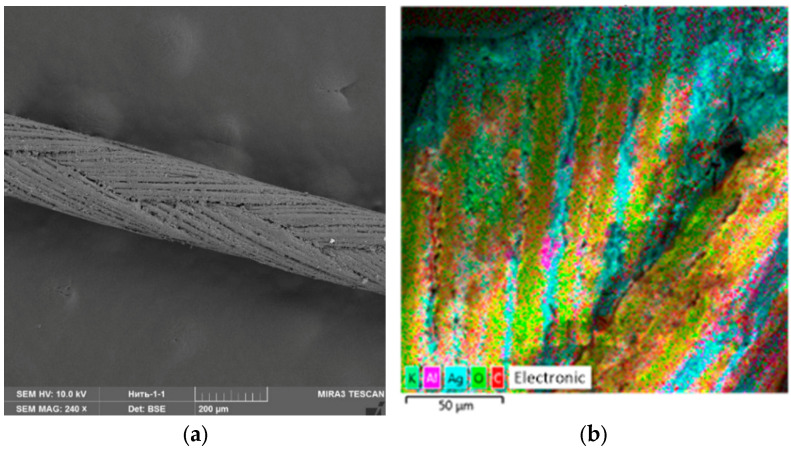

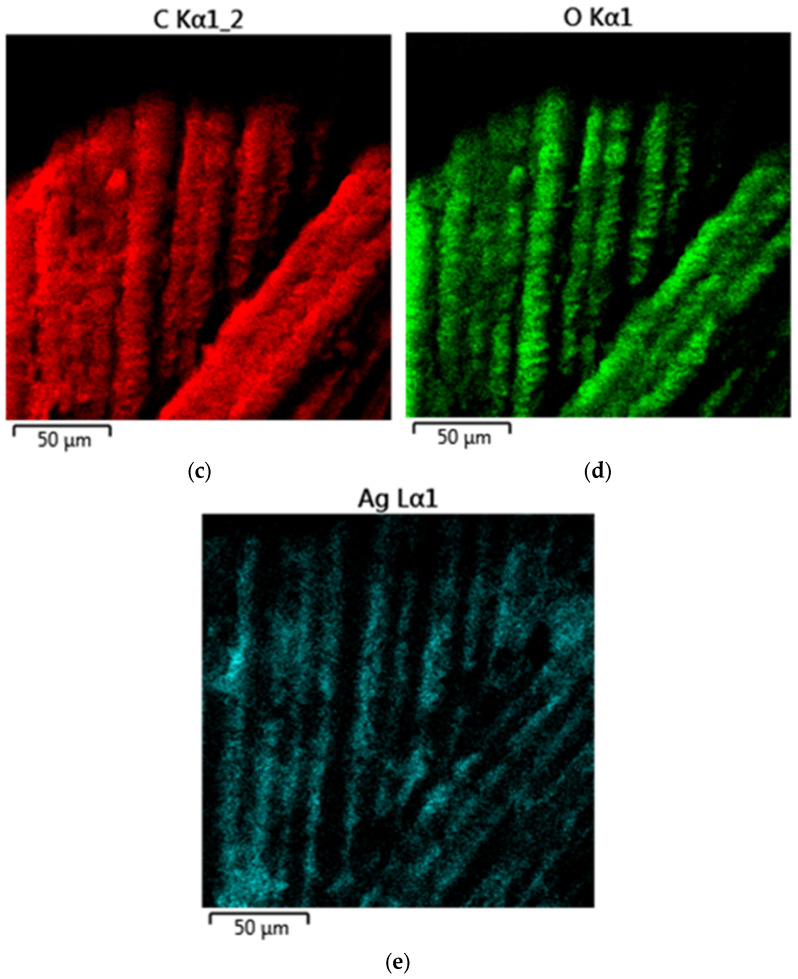
A sample of suture material treated by Ag NPs: (**a**)—SEM micrograph, (**b**)—multilayer map of EDS, (**c**)—elemental map of EDS (C), (**d**)—elemental map of EDS (O) and (**e**)—elemental map of EDS (Ag).

For the experiment, we treated the suture material with Ag NPs and the oxidized Ag NPs. Figure 17, Figure 18 and Figure 19 show SEM micrographs and multilayer and elemental maps of an energy dispersion analysis (EDS) of the surface of suture materials samples.

An analysis of Figure 17, Figure 18 and Figure 19 showed that Ag NPs and oxidized Ag NPs formed a homogeneous and uniform layer on the surface of the suture material.

In the next stage, we carried out in vivo studies of suture material samples treated by Ag NPs and oxidized Ag NPs. The studies were conducted on Californian breed rabbits (*Sylvilagus bachmani*).

**Figure 20:**
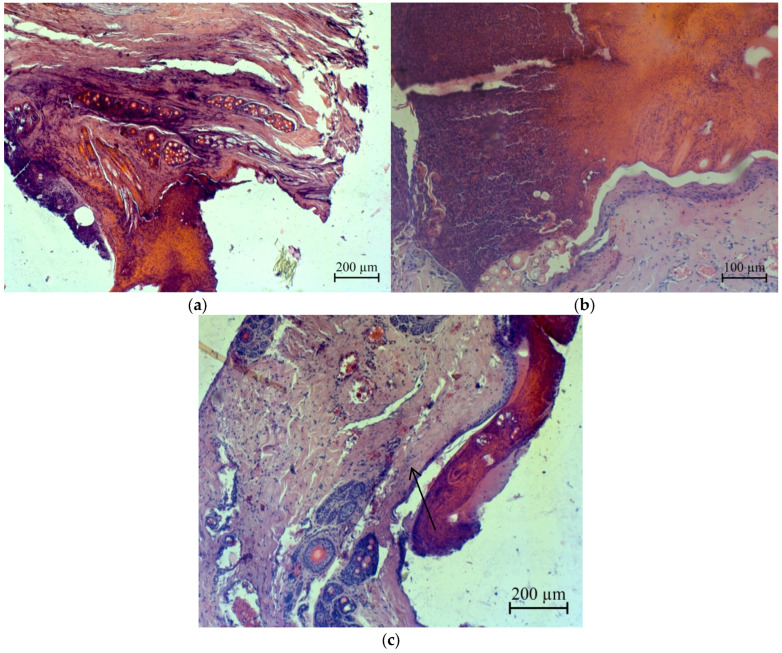
Micrographs of histological sections of postoperative wounds of laboratory animals: (**a**)—the first group, (**b**)—the second group and (**c**)—the third group.

A histological examination of postoperative wound sections on rabbit skin revealed that the pathomorphological changes were identical in all three areas. On the surface of the postoperative wound, a scab is visible (marked by arrow), consisting of clotted blood and tissue fragments, which constitute a homogeneous, oxyphilic mass with insignificant basophilic inclusions, completely covering the wound surface and the skin surface surrounding the wound surface (Figure 20).

**Figure 21:**
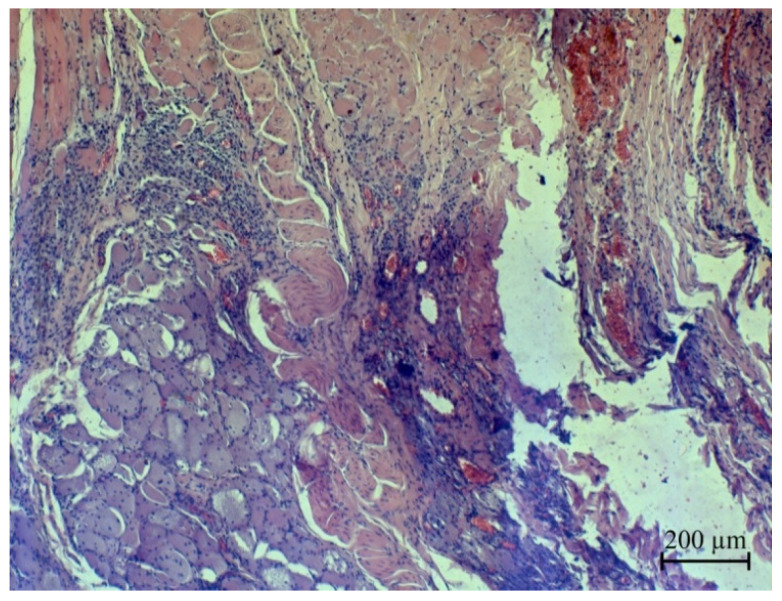
Accumulation of cellular infiltrate under the scab of animals of the first group.

Under the scab and in the tissues surrounding the wound canal, we observed extensive cellular infiltrates, consisting mainly of macrophages, lymphocytes and new connective tissue cells. The width of extensive cellular infiltrates was approximately 300–350 μm in animals of all experimental groups (Figure 21).

**Figure 22:**
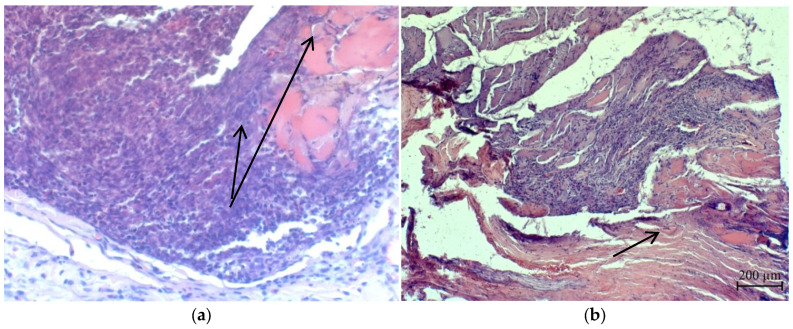
Accumulation of cellular infiltrate under the scab: focal necrosis of muscle fibers (indicated by the arrow): (**a**)—the first group and (**b**)—the second group.

In single skeletal muscles located directly adjacent to the wound canal, pathomorphological changes characteristic of focal necrosis were detected (Figure 22).

**Figure 23:**
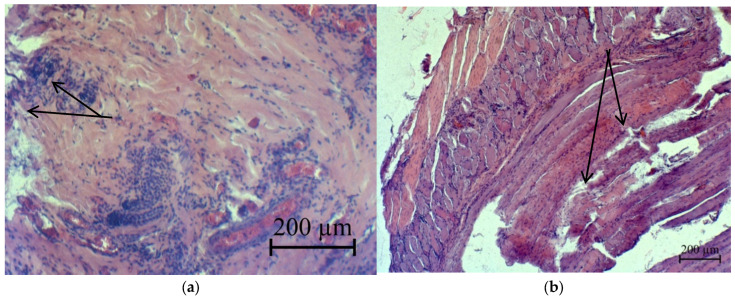
Accumulation of cellular infiltrate between muscle fibers: focal necrosis of muscle fibers (indicated by the arrow): (**a**)—the second group and (**b**)—the third group.

Necrotized skeletal muscles are slightly thickened, streakless and homogeneous, representing a homogeneous oxyphilic mass. In deeper lying tissues from extensive cellular infiltration at a depth of 400.0–500.0 μm, cellular infiltrates consisting mainly of lymphocytes and single macrophages were insignificant and were located mainly only around blood vessels in the form of couplings (Figure 23). Changes in blood vessels are characteristic of arterial hyperemia.

**Figure 24:**
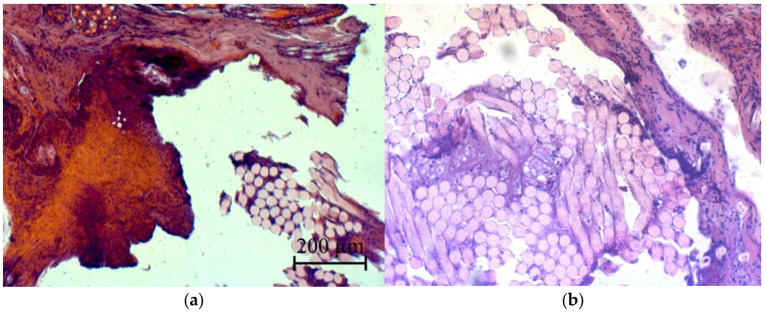
Micrographs of histological sections of postoperative wounds of laboratory animals (fragments of suture material): (**a**)—the second group and (**b**)—the third group.

There was no evidence of an allergic reaction of the body at the sites where the suture material was detected. No extensive cellular infiltrates were detected around the suture material, and eosinophils were characteristic of an allergic reaction of the body to foreign bodies (Figure 24).

## 4. Conclusions

In of our research, we obtained Ag NPs stabilized with PVP ranging in size from 70 to 110 nm, which have good crystallinity and an anisotropic structure. We established for the first time that the interaction of PVP molecules with the surface of Ag NPs is through the formation of a N-Ag chemical bond. In our research, it was shown that Ag NPs have a high aggregation stability in the pH range of 2–12. With an increase in the ionic strength of the solution, we observed a decrease in the intensity of the surface plasmon resonance band, which is associated with an increase in the size of Ag NPs due to electrostatic coagulation under the action of Cl^−^ ions.

To the best of our knowledge, this is the first time we have studied the oxidation process of Ag NPs. We established that for molar ratios Ag:H_2_O_2_ = 1:1 and 1:5, dependences of changes in the intensity, position and half-width of the absorption band of the plasmon resonance are rectilinear, and complete discoloration after 80 min of exposure was not observed. At molar ratios Ag:H_2_O_2_ = 1:10, 1:15 and 1:20, dependences of the intensity of the absorption band of the plasmon resonance of Ag NPs took the form of exponents, and complete discoloration of the sols was observed after 20 min, 30 min and 40 min, respectively.

In vivo studies of oxidized Ag NPs showed that it belongs to toxicity class III (a moderately dangerous substance) and the third group according to the degree of accumulation. The analysis of SEM micrographs and EDS multilayer and elemental maps showed that Ag NPs and oxidized Ag NPs formed a homogeneous and uniform layer on the surface of the suture material. During the in vivo experiment, we found that when the suture material was treated with Ag NPs and oxidized Ag NPs, no signs of allergic reactions from the bodies of laboratory animals were detected.

The data obtained by us provide a fundamental and applied basis for the use of Ag NPs and oxidized Ag NPs as an antibacterial agent for the treatment of suture material in veterinary and medical practice.

## Figures and Tables

**Table 1:** Results of quantum chemical calculations of the Ag-PVP molecular system.

Index	E, kcal/mol	HOMO	LUMO	*η*
Interactions of a Ag atom with a PVP monomer unit through oxygen	−5532.8331	−0.214	−0.068	0.073
Interactions of a Ag atom with a PVP monomer unit through nitrogen	−5533.5474	−0.288	−0.118	0.085

**Table 2:** Acute toxicity of oxidized Ag NPs stabilized with PVP with a single oral administration.

Type of Introduction	Toxicity Parameters					SLD_50_
	MSD	LD_16_	LD_50_	LD_84_	LD_100_	
Preoral	4408.0	4422.74	4400.0	4463.4	4500.0	±0.644

## Data Availability

All data are available upon request to the corresponding author.

## Supplementary Materials

The following supporting information can be downloaded at: https://www.mdpi.com/article/10.3390/mi13071105/s1, Figure S1: Absorption spectra of the mixture of Ag NPs and oxidizer in the ratio: a—Ag:H_2_O_2_ = 1:5; b—Ag:H_2_O_2_ = 1:10; c—Ag:H_2_O_2_ = 1:15; d—Ag:H_2_O_2_ = 1:20; Figure S2: Kinetic dependences of the change of half-width at the half-height of the absorption band of the plasmon resonance of Ag NPs during oxidation with H_2_O_2_.
