# Supplementary material for: Surface-Oxidized Polymer-Stabilized Silver Nanoparticles as a Covering Component of Suture Materials
Source: Micromachines (Basel). 2022 Jul 14;13(7):1105. doi: 10.3390/mi13071105 (PMC9323226; doi:10.3390/mi13071105)
Supplement: Supplementary file 1 [file micromachines-13-01105-s001.zip › micromachines-1814694-supplementary.pdf]

micromachines

Article

Supplementary Materials

Surface-Oxidized Polymer-Stabilized Silver Nanoparticles as a Covering Component of Suture Materials

Andrey Vladimirovich Blinov [1], Andrey Ashotovich Nagdalian [1,2,*], Sergey Nikolaevich Povetkin [1], Alexey Alekseevich Gvozdenko [1], Marina Nikolaevna Verevkina [3], Igor Vladimirovich Rzhepakovsky [1], Mariya Sergeevna Lopteva [4], David Guramievich Maglakelidze [1], Tatyana Semenovna Kataeva [5], Anastasiya Aleksandrovna Blinova [1], Alexey Borisovich Golik [1], Galina Vladimirovna Osipchuk [6] and Mohammad Ali Shariati [7]

1 Department of Physics and Technology of Nanostructures and Materials, North Caucasus Federal University, 355017 Stavropol, Russia; ablinov@ncfu.ru (A.V.B.); d22003807-help@mail.ru (S.N.P.); gvozdenko.1999a@gmail.com (A.A.G.); 78igorr@mail.ru (I.V.R.); ogoniock2015@mail.ru (D.G.M.); nastya_bogdanova_88@mail.ru (A.A.B.); lexgooldman@gmail.com (A.B.G.)
2 Saint Petersburg State Agrarian University, 196601 Saint Petersburg, Russia
3 Department of Parasitology and Veterinary Examination, Anatomy and Pathanatomy Named after Professor S. N. Nikolsky, Stavropol State Agrarian University, 355017 Stavropol, Russia; serenkiy7@yandex.ru
4 Veterinary Medicine Laboratory of the Animal Husbandry and Veterinary Medicine Department, North Caucasus Federal Agrarian Research Centre, 356241 Mikhailovsk, Russia; zmejka-2007@mail.ru
5 Department of Parasitology, Veterinary and Sanitary Expertise, Faculty of Veterinary Medicine, Kuban State Agrarian University Named after I. T. Trubilin, 350044 Krasnodar, Russia; cluster26@yandex.ru
6 Laboratory of Embryos Reproduction and Transplantation, Scientific-Practical Institute of Biotechnologies in Animal Science and Veterinary Medicine, 249053 Maksimovka Village, Moldova; galadok@rambler.ru
7 Department of Scientific Research, K.G. Razumovsky Moscow State University of Technologies and Management (The First Cossack University), 109004 Moscow, Russia; shariatymohammada-li@gmail.com
* Correspondence: geniando@yandex.ru

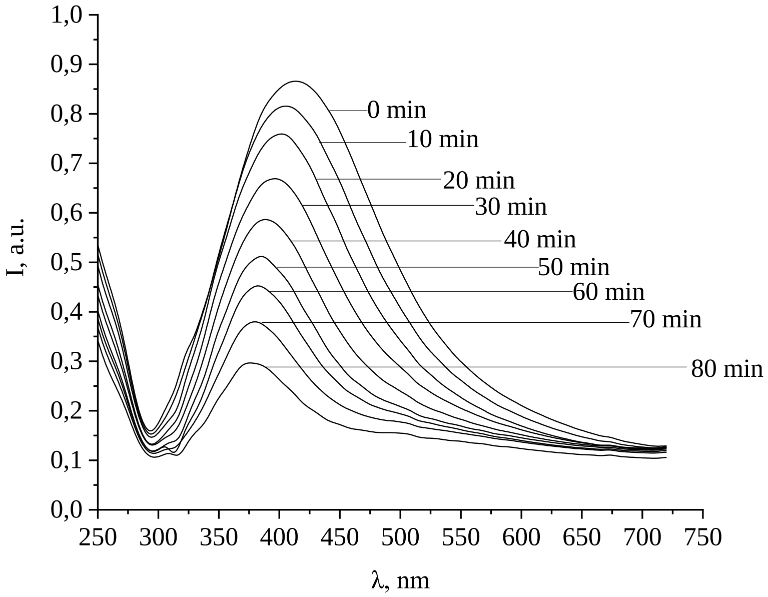

a

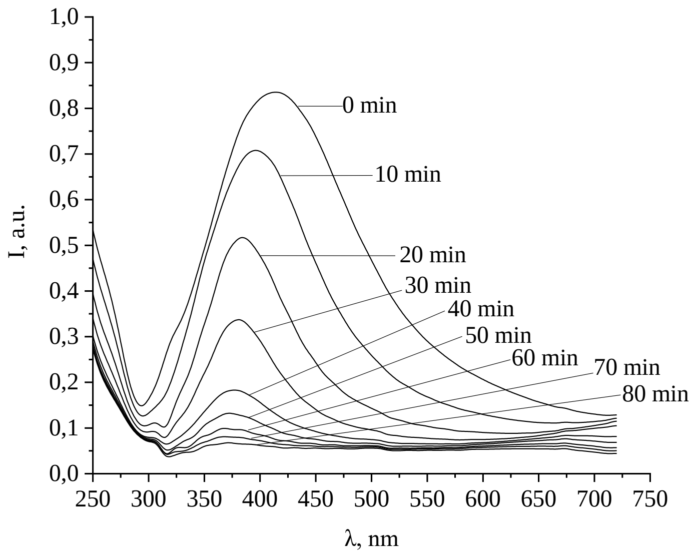
1,0
0,9
0,8
0,7
0,6
0,5
0,4
0,3
0,2
0,1
0,0
I, a.u.
0 min
10 min
20 min
30 min
40 min
50 min
60 min
70 min
80 min
250 300 350 400 450 500 550 600 650 700 750
λ, nm

b

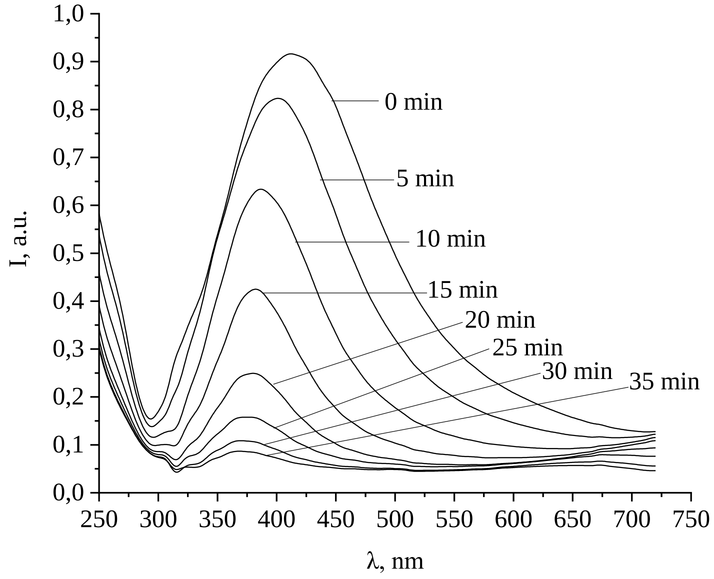
1,0
0,9
0,8
0,7
0,6
0,5
0,4
0,3
0,2
0,1
0,0
I, a.u.
0 min
5 min
10 min
15 min
20 min
25 min
30 min
35 min
250 300 350 400 450 500 550 600 650 700 750
λ, nm

c

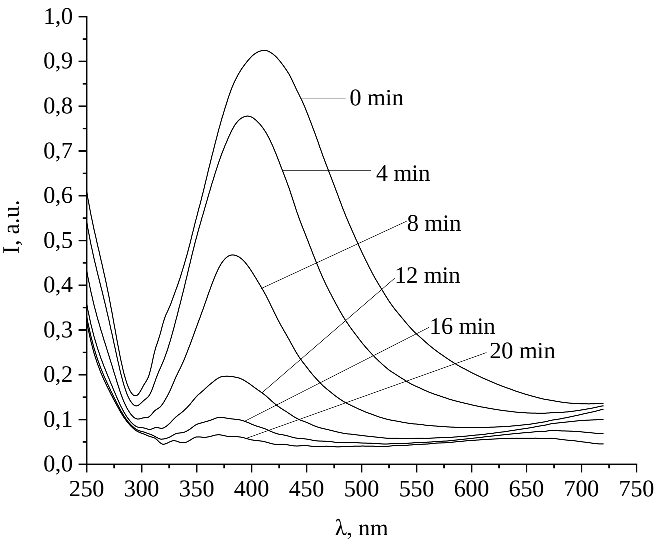

d

Figure S1. Absorption spectra of the mixture of Ag NPs and oxidizer in the ratio: a – $Ag:H_2O_2 = 1:5$; b – $Ag:H_2O_2 = 1:10$; c – $Ag:H_2O_2 = 1:15$; d – $Ag:H_2O_2 = 1:20$.

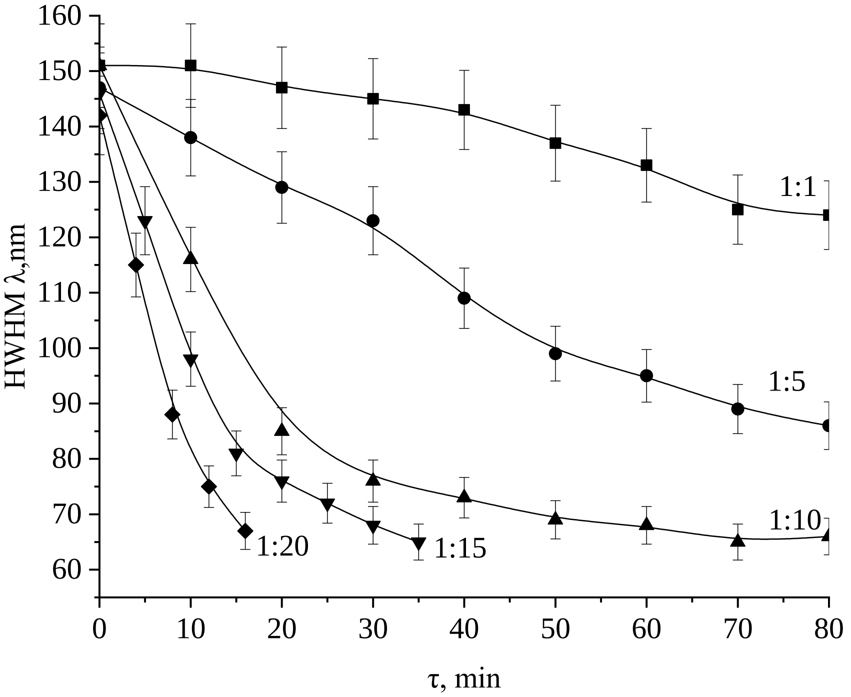

Figure S2. Kinetic dependences of the change of half-width at the half-height of the absorption band of the plasmon resonance of Ag NPs during oxidation with H_2O_2.
